# Variation in fatty acid composition of the bigeye snapper *Lutjanus lutjanus* collected in coral reef habitats of the Malaysian South China Sea

**DOI:** 10.1186/s40709-015-0027-2

**Published:** 2015-04-18

**Authors:** Takaomi Arai, Razikin Amalina, Zainudin Bachok

**Affiliations:** Institute of Oceanography and Environment, Universiti Malaysia Terengganu, 21030 Kuala Terengganu Terengganu, Malaysia; School of Marine Science and Environment, Universiti Malaysia Terengganu, 21030 Kuala Terengganu Terengganu, Malaysia

**Keywords:** Coral fish, Diet, Habitat, Lutjanidae, Migration, South China Sea

## Abstract

**Background:**

In order to understand trophic ecology, habitat use and migration of coral reef fish, fatty acid composition and levels were examined in the bigeye snapper *Lutjanus lutjanus* collected in the Malaysian South China Sea.

**Results:**

Proportions of saturated fatty acids (SAFA) ranged from 55.0% to 66.5%, with the highest proportions in fatty acids, the second highest was monounsaturated fatty acids (MUFA) ranged from 30.7% to 40.2% while the proportion of polyunsaturated fatty acids (PUFA) was the lowest ranged from 2.8% to 4.8%. Palmitic acid (16:0) was the most common in SAFA, oleic acid (C18:1ω9c) was the dominant in MUFA and linolenic acid (C18:3n3) showed the highest value in PUFA. Fatty acid concentrations, especially in SAFA and MUFA, increased with fish growth, suggesting diet and habitat shifts during the fish life history. Most of the fish had more than 1 of EPA: DHA ratio, which suggested that diets of *L. lutjanus* tended to be higher trophic organisms such as zooplankton and crustacean in coral ecosystem.

**Conclusions:**

The diet shift revealed by the composition and levels of the fatty acid profile revealed potential pattern in the habitat use and migration scale in coral reef environment of *L. lutjanus*.

## Background

Southeast Asia is recognised as the global centre for coral reefs, both in terms of extent and species diversity. An estimated 34% of the Earth’s coral reefs are located in the seas of Southeast Asia, which occupy only 2.5% of the Earth’s total sea surface [1]. Ecologically, the coral reefs of the South China Sea are sources of larvae and juveniles for many commercially important reef fish. 710 species of marine fishes from the Malaysian water and their adjacent seas are reported [2]. Furthermore, Ambak *et al*. [3] and Chong *et al*. [4] listed 2243 and 1951 fish species, respectively, in Malaysian water. Thus, Malaysia has the one of the highest and richest diversity of fish in the world [5]. Although several information regarding taxonomy and distribution in coral fish species is available in Malaysian water, few studies have been done for their life history, ecology and reproduction compared to other coral reef areas.

Information on the diet and trophic position of an animal is fundamental issue to understand its life history. Recently, signature of fatty acid analysis has been increasingly used to study the diet of a number of marine species [6-11]. The use of fatty acids as trophic biomarkers is based on that many fatty acids in the marine environment are characteristic of specific groups of marine organisms [7,9-11]. Fatty acids can generally not be synthesised in higher trophic levels and are incorporated into the consumers’ tissues with minimal modification, thus retaining signatures of their dietary origin [12]. Once fatty acid patterns are established for prey, they can be used to trace food webs and diets of higher predators. Thus, fatty acid analysis could support to resolve trophic interactions in marine ecosystems.

Bigeye snapper *Lutjanus lutjanus* is a commercially important coral reef fish species, geographically distributed in the Indo-West Pacific from the Solomon Islands to East Africa and from Australia to southern Japan [13]. The species is found in large schools of more than 100 individuals with other *Lutjanus* species [13,14]. *Lutjanus lutjanus* can reach a maximum of 30 cm in total length but generally grows up to 19 cm [13,15]. As a carnivorous species, preys of *Lutjanus* species are believed to be small fishes and crustaceans [13]. However, diet and feeding ecology of the fish is scarcely understood.

In the present study, fatty acid analyses were used to investigate the trophic ecology of the bigeye snapper *Lutjanus lutjanus* collected in Malaysian South China Sea. To understand the trophic position in accordance with the growth, fatty acid signatures were compared using various size class samples. These fatty acid data will also be useful in food web studies to understand coral reef ecosystems.

## Results

### Biological characteristics

Biological characteristics such as TL and BW of the bigeye snapper *Lutjanus lutjanus* ranged from 13.2 cm to 19.5 cm and from 30.6 g to 109 g, respectively (Table 1). Significant differences were found among three size classes in TL (*p* < 0.0001), BW (*p* < 0.0001) and liver weight (LW) (*p* < 0.05). No significant differences were found in GSI among three size classes, suggesting same maturation stage in all fishes (Table 1).

**Table 1:** **Biological information of** ***Lutjanus lutjanus*** **collected in the Bidong Island, Malaysian South China Sea**

	**Total length (cm)**		**Body weight (g)**		**Liver weight (g)**		**GSI**		
**Size class**	**Mean ± SD**	**Range**	**Mean ± SD**	**Range**	**Mean ± SD**	**Range**	**Mean ± SD**	**Range**	**N**
Small	14.6 ± 0.73	13.2 - 15.6	43.6 ± 6.99	30.6 - 54.0	0.244 ± 0.089	0.110 - 0.405	0.28 ± 0.12	0 - 0.42	12
Medium	16.3 ± 0.64	15.2 - 18.5	60.7 ± 5.07	48.1 - 67.9	0.325 ± 0.090	0.195 - 0.559	0.24 ± 0.15	0 - 0.52	21
Large	18.1 ± 0.75	17.0 - 19.5	79.0 ± 13.3	62.8 - 109	0.509 ± 0.159	0.329 - 0.868	0.18 ± 0.18	0 - 0.46	12

N: total number of specimens.

Stomach content for each fish was observed for ten randomly chosen samples. However, stomach content for each fish could not identify prey organisms under macro- and micro-observations. Thus, we did not conduct stomach content observations for other 35 fishes.

### Fatty acid composition

Proportions of saturated fatty acids (SAFA) ranged from 55.0% to 66.5%, with the highest proportions in fatty acids (Table 2). Palmitic acid (16:0) was the most common saturated fatty acid and ranged from 39.9% to 52.4% (Table 2) followed by C18:0 and C14:0. Significant differences in ∑SAFA were found between small fishes (SFs) and medium fishes (MFs) (*p* < 0.005) and between SFs and large fishes (LFs) (*p* < 0.05); however no significant difference was found between MFs and LFs (*p* > 0.05).

**Table 2:** **Fatty acid composition (mean ± SD) in livers of** ***Lutjanus lutjanus*** **collected in the Bidong Island, Malaysian South China Sea**

**Fatty acids**	**Small (n = 3)**	**Medium (n = 7)**	**Large (n = 6)**
SAFA			
C14:0	7.3 ± 2.4	4.4 ± 0.6	4.5 ± 0.7
C16:0	39.9 ± 5.9^a^	52.4 ± 6.4^b^	48.6 ± 8.2^b^
C18:0	7.6 ± 2.2	9.4 ± 5.6	7.4 ± 4.9
C20:0	0.2 ± 0.1	0.3 ± 0.2	0.3 ± 0.2
**∑SAFA**	**55.0 ± 1.6** ^a^	**66.5 ± 7.4** ^b^	**60.8 ± 9.2** ^b^
MUFA			
C16:1	11.9 ± 4.9	8.9 ± 1.4	9.6 ± 2.6
C17:1	1.2 ± 0.4	1.1 ± 0.4	1.4 ± 0.4
C18:1ω9c	13.2 ± 1.3	12.0 ± 3.0	15.1 ± 6.4
C18:1ω9t	13.7 ± 3.5	7.6 ± 5.8	9.5 ± 6.0
C20:1	0.1 ± 0.1^a^	1.1 ± 1.2^b^	0.8 ± 0.4^b^
**∑MUFA**	**40.2 ± 2.2**	**30.7 ± 8.0**	**36.4 ± 9.3**
PUFA			
C18:3n6	0.04 ± 0.1	0.1 ± 0.1	0.1 ± 0.1
C18:3n3	2.9 ± 1.6	1.5 ± 0.6	1.1 ± 0.3
C20:3n3	0.03 ± 0.05	0.02 ± 0.04	0.01 ± 0.03
C20:5n3 (EPA)	1.4 ± 0.4	0.6 ± 0.2	0.6 ± 0.3
C22:6n3 (DHA)	0.5 ± 0.7	0.5 ± 0.8	1.1 ± 0.8
EPA/DHA	12.3 ± 11.7	2.9 ± 1.7	1.0 ± 1.2
**∑PUFA**	**4.8 ± 1.4**	**2.8 ± 1.1**	**2.8 ± 1.1**

^a,b^Means in the same row with different superscript differ significantly (*p* < 0.05).

Monounsaturated fatty acids (MUFA), which were the second dominant, ranged from 30.7% to 40.2% (Table 2). Of all MUFA, oleic acid (C18:1ω9c) was the dominant MUFA for all size classes (Table 2). No significant differences were found between all size classes (*p* > 0.05), except for between small fishes and medium and large fishes (*p* < 0.05) in C20:1.

The proportion of polyunsaturated fatty acids (PUFA) was accordingly low; the mean value ranged from 2.8% to 4.8% (Table 2). Linolenic acid was highest ranging from 1.1% to 2.9%, followed by EPA (C20:5n3) and DHA (C22:6n3) (Table 2). No significant differences were found between all size classes (*p* > 0.05).

EPA/DHA ratios in small, medium and large fish were 12.3 ± 11.7 (mean ± SD) ranging from 0.7 to 24, 2.9 ± 1.7 ranging from 0.3 to 5.9 and 1.0 ± 1.2 ranging from 0.3 to 3.3, respectively. No significant differences were found between all size classes (*p* > 0.05).

### Fatty acid concentrations

**Figure 1:**
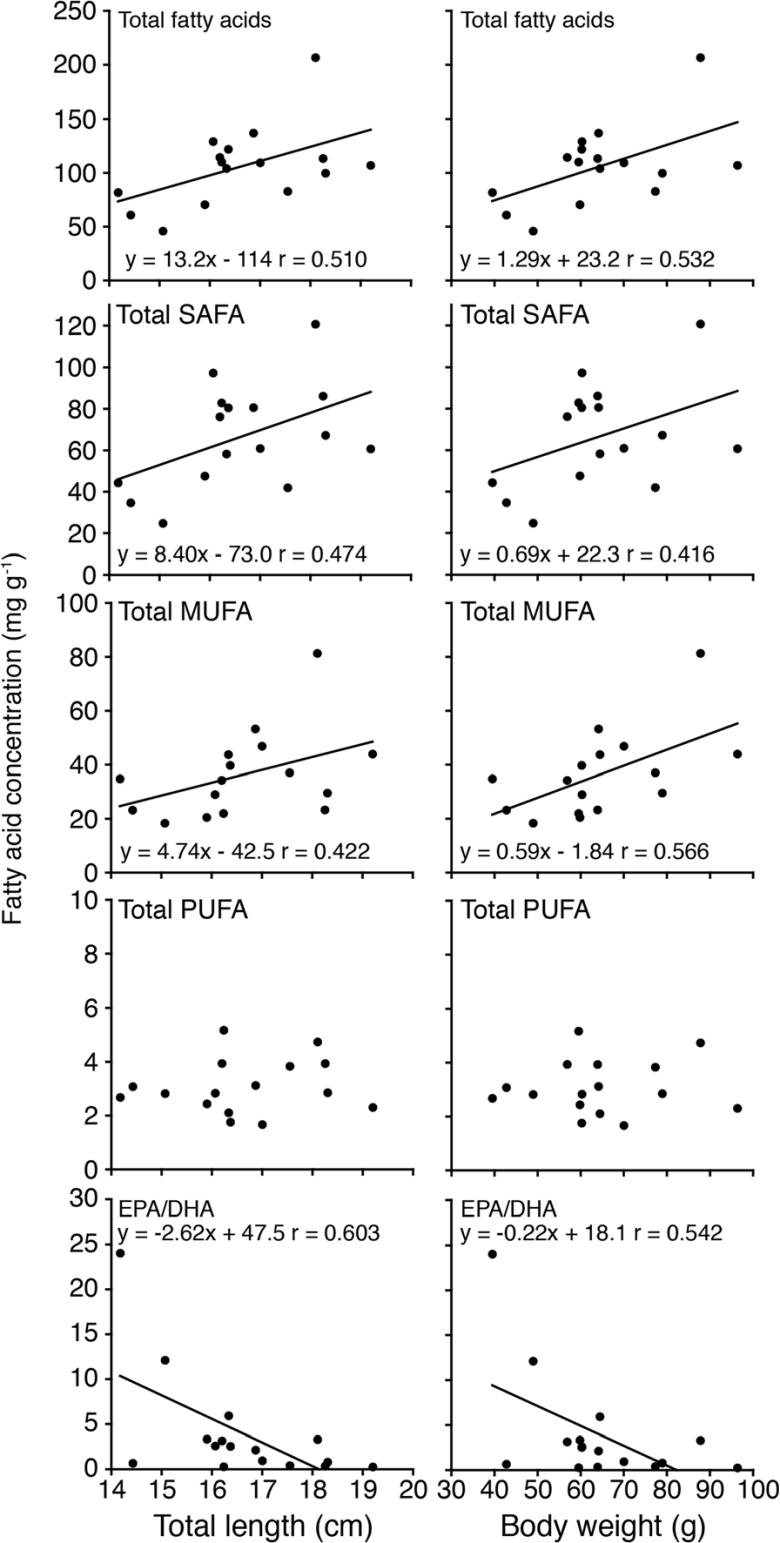
Relationship between fatty acid levels such as ∑FA, ∑SAFA, ∑MUFA and ∑PUFA and EPA/DHA ratio and total length (TL) and body weight (BW) in the livers of the bigeye snapper *Lutjanus lutjanus* collected at the Bidong Island in Malaysian South China Sea.

**Figure 2:**
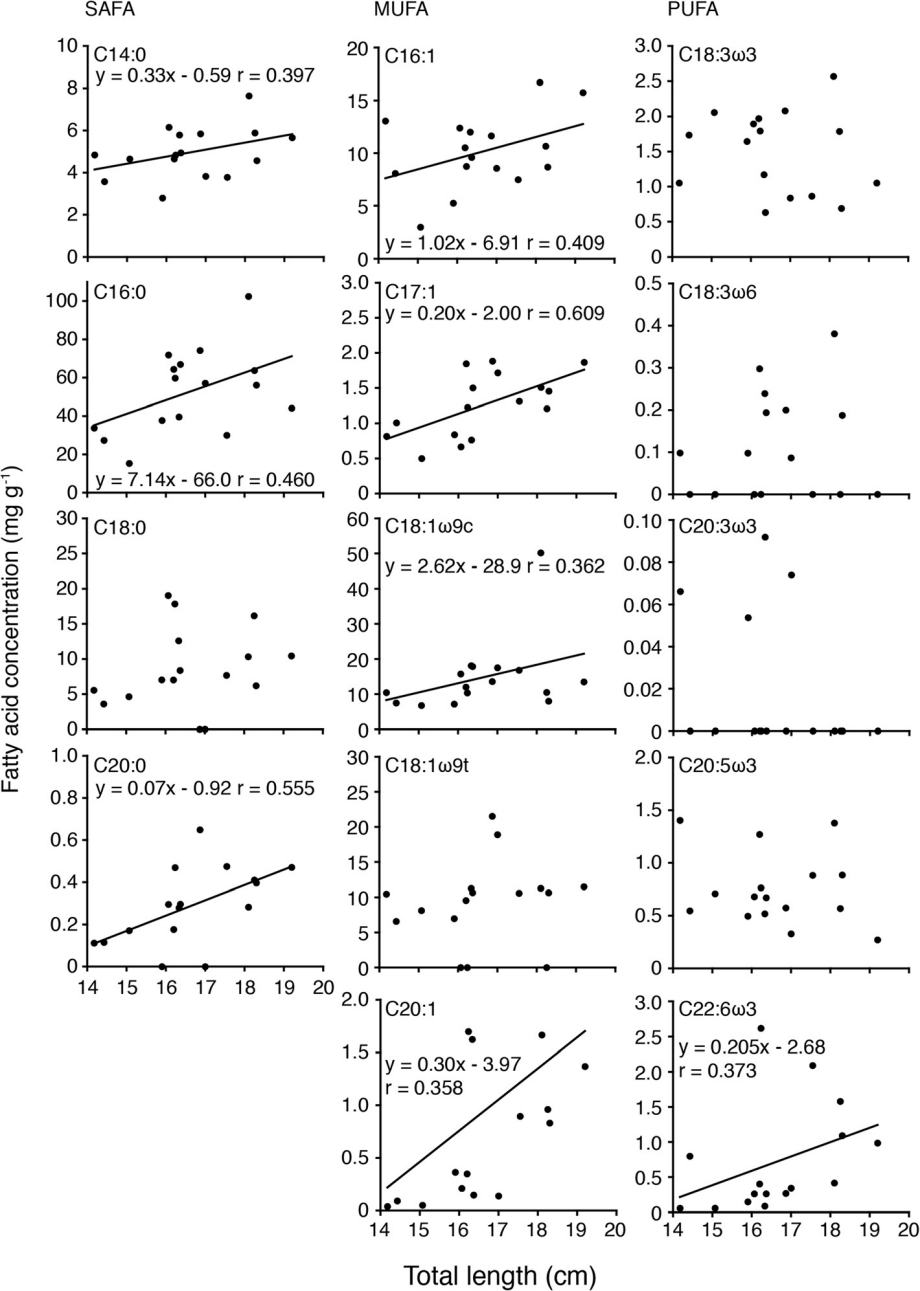
Relationship between each fatty acid level and TL in the livers of the bigeye snapper *Lutjanus lutjanus* collected at the Bidong Island in Malaysian South China Sea.

**Figure 3:**
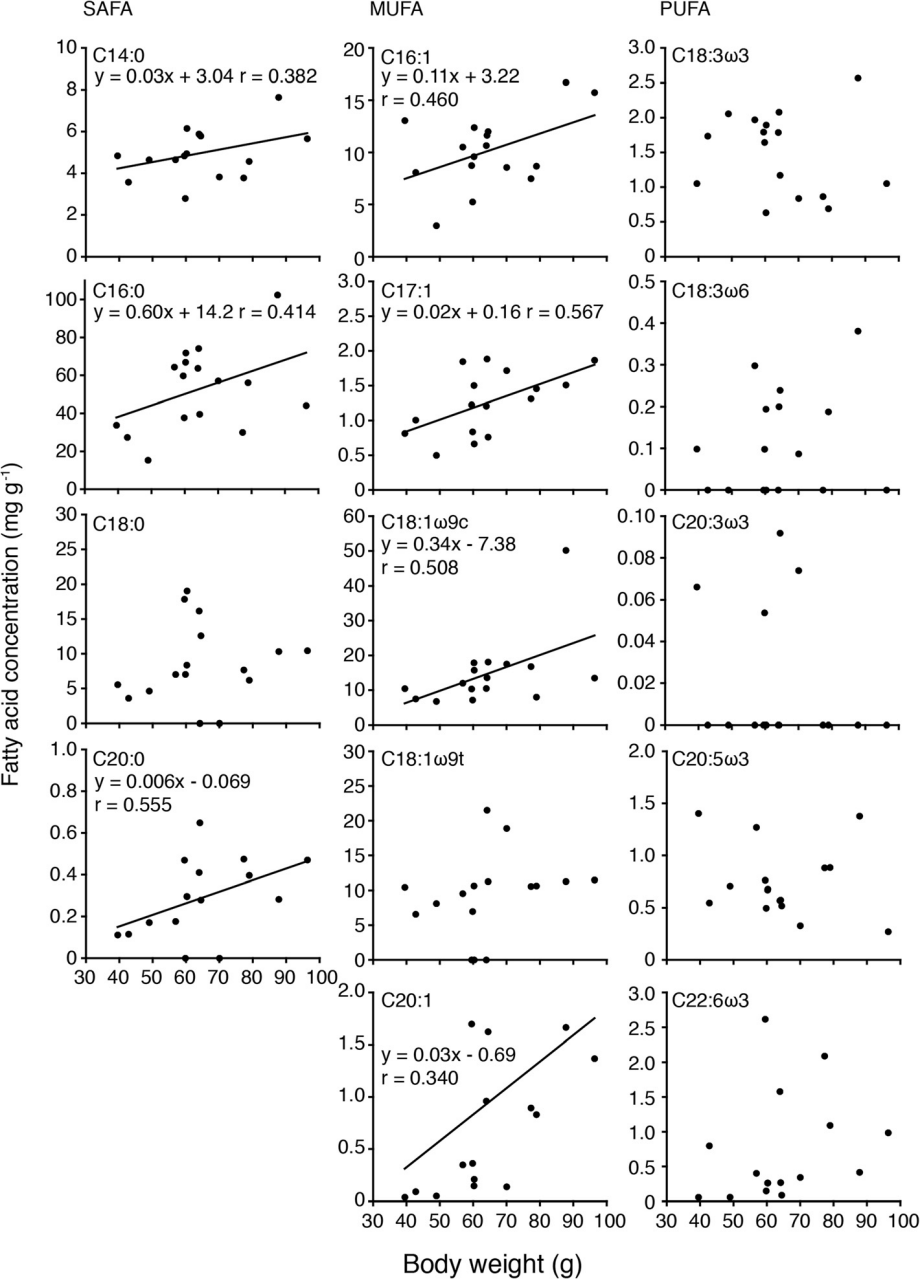
Relationship between each fatty acid level and BW in the livers of the bigeye snapper *Lutjanus lutjanus* collected at the Bidong Island in Malaysian South China Sea.

Fatty acid concentrations increased with fish size (Figures 1, 2 and 3), although correlation coefficients were not “strong” for all relationships. Close positive relationships were found between ∑FA, ∑SAFA and ∑MUFA and TL and BW (*p* < 0.05-0.0001), however no close relationship was found between ∑PUFA and TL and BW (*p* > 0.05) (Figure 1).

**Figure 4:**
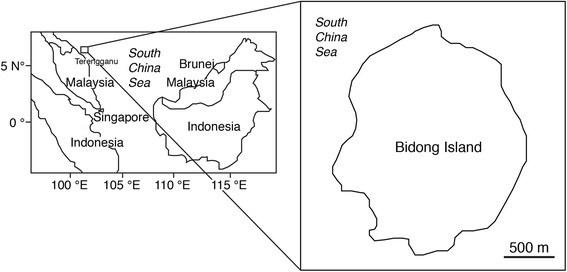
Map showing the location of the study site at the Bidong Island in Malaysian South China Sea, off the Terengganu State in the east coast of Peninsula Malaysia.

In SAFA, close positive relationships were found between C14:0, C16:0 and C20:0 and TL and BW (*p* < 0.05-0.0005), however no close relationship was found between ∑PUFA and TL and BW (*p* > 0.05) (Figures 2 and 3). Concentrations of C16:1, C17:1, C18:1ω9c and C20:1 in MUFA significantly increased with fish size (*p* < 0.05-0.0001), however no correlations were found between C18:1ω9t and TL and BW (*p* > 0.05) (Figures 2 and 4). No significant relationships existed between each fatty acid and fish size in PUFA (*p* > 0.05), except for a relationship between C22:6ω3 and TL (*p* < 0.05) (Figures 2 and 3).

Close negative relationships were found between EPA/DHA ratios and TL and BW (*p* < 0.005, Figures 2 and 4) suggesting the fish diets are different from their size.

## Discussion

It is noteworthy that fatty acid composition and levels were different depending on the growth stage; both parameters were increased as fish became larger (Figures 1, 2 and 3). Differences in individual fatty acid profiles in relation to development [16,17], food habits [6-11] and habitat use [18-20], temperature [18] and salinity [20,21] have been reported in different fish species. However, differences in fatty acid composition and levels in relation to body size in wild fish species have not been well established. Although the mechanism of lipid deposition in the liver of fish fed diets was still uncertain, fatty acid synthesis was regulated by liver X receptor suggesting the profiles of the liver reflected diets of fish [22]. It is likely that such differences are caused by differences in the diet, behavior and migration of *L. lutjanus* accompanying the growth. The role of seagrass beds and mangroves as nursery habitats for some fish species has received considerable attention as a link with adjacent coral reef or offshore habitats [23-25]. The spatial size distribution of snappers (Lutjanidae) has previously indicated the separation of juveniles in nursery habitats from the adults on the coral reef [26]. In the ontogeny of Lutjanidae, a major change in diet occurred at a size-class that corresponded to the size at which these individuals were first observed on the coral reef [26]. The diet shifts of snapper (Lutjanidae) species that inhabit seagrass beds and mangroves have been reported during their juvenile and sub-adult stages [26-28]. In the case of Lutjanidae, the size at the critical diet shift corresponded to the smallest size at which these species were observed on the adjacent reef [26]. The major diet shift and diet change are suggested to play an important role in determining their migration patterns [26]. These findings suggest that differences in fatty acid profile during growth found in the present study might correspond to the diet and habitat shifts in *L. lutjanus*.

SAFA was the most abundant fatty acids and the palmitic SAFA showed highest values among all fatty acids (Table 2). The second most abundant SAFA was stearic acid. These two SAFAs have been reported to have the highest concentrations in other fish species [29,30], *Acetes* [31] and copepods [32]. The predominance of both fatty acids has been attributed to their use as a major source of energy for metabolism and growth [33]. Fishes from warm waters tend to show high levels of palmitic and stearic acids compared to those from cold waters [34]. This difference is due to metabolic differences between cold and warm water species, because these fatty acids are not usually subject to differences in diet [34]. The bigeye snapper *Lutjanus lutjanus* was collected in the South China Sea in tropical waters in the present study, and thus the fish might have higher palmitic and stearic acid levels.

MUFA was the second most abundant fatty acid, with highest values for oleic acid (Table 2). This is in agreement with findings in copepods [35], *Acetes* [31] and fish fatty acid profiles [29,30,34]. Oleic MUFA is naturally occurring in large concentrations in many marine organisms, which can also synthesise this MUFA *de novo* [33]. High proportions of MUFAs of marine predators are generally derived from marine zooplankton in particular calanoid copepods such as *Calanoides acutus* and *Calanus propinquus* in Antarctic waters [36,37]. Recently, damselfish species *Abudefduf bengalensis* and *A. sexfasciatus* collected in coral reef are of the South China Sea, showed the high proportion of MUFAs [10,11,38]. These fishes have been suggested to consume marine zooplankton [10,11,38]. In the present study, we did not conduct fatty acid analyses for potential prey organisms. Nevertheless, the higher level of MUFAs found in *Lutjanus lutjanus* suggest that the fish might feed copepod as one of potential prey organism during the life history.

EPA and DHA showed the highest levels among PUFA (Table 2). These two PUFAs are also known as highly unsaturated fatty acids. EPA:DHA ratios ranged from 1.0 to 12.3 in *Lutjanus lutjanus* (Table 2). EPA:DHA ratios of copepods *Acartia erythraea*, shrimps *Lucifer penicillifer* and *Acetes intermedius* and *A. erythraeus* and pelagic fishes such as anchovy, sardine and scad ranged from 0.1 to 1.2 collected in the Mindanao Sea, Philippines [39] where is geographically close to the South China Sea. The ratios suggest that *Lutjanus lutjanus* might feed copepods, shrimps and small pelagic fishes in coral ecosystem.

## Conclusions

Fatty acid signature has been increasingly used to study the diet of a number of marine species. The present study suggests that diets of the coral fish species *Lutjanus lutjanus* changed in accordance with growth. Furthermore, differences in fatty acid profiles might not just be considered with respect to the diets, but might be based on the habitat and migration. Further studies are needed to study for various organisms in coral ecosystem using fatty acid signature for understanding life history and ecology details in the coral fish species.

## Methods

### Fish

All specimens of the bigeye snapper *Lutjanus lutjanus* were collected at the Bidong Island in the South China Sea, Malaysia (05°37′12″ Ν, 103°04′12″ Ε) between 27 and 28 October 2013 (Figure 4). Bidong Island is located off Terengganu State on the east coast of Peninsular Malaysia, known for its well-developed coral reef ecosystems that support a variety of coral and rocky reef associated fishes [40]. All fishes were collected by means of fish traps and hook and line. After collecting, all fishes were immediately stored in ice chest, brought back to laboratory, were kept in −20°C freezer. Fatty acid analyses were conducted within one month after sampling. A total of 45 fish samples were measured in total length (TL), body weight (BW), and each fish was dissected in order to determine liver and gonad weights (Table 1). In the present study, fishes were categorised as either small, medium or large according to their sizes (Table 1). Stomach for each fish was dissected for the content analyses. Gonadosomatic index (GSI) for each fish was calculated with the formula

$$ GSI = GW\ B{W}^{-1} \times 100 $$

### Fatty acid analysis

Liver samples of *Lutjanus lutjanus* were analysed for fatty acid composition following the one step method [9-11]. Liver samples were combined to form pooled tissue samples for each size group. The sample sizes of small, medium and large groups were three, seven and six, respectively (Table 2). Each liver sample was mixed with 4 ml of hexane (Merck, Germany) and 1 ml of internal standard solution in a 50 ml centrifuge tube. After adding 2 ml of 14% BF3 in methanol, the tube was flushed with nitrogen gas. The capped tube was heated on a hot plate at 100°C for 120 min. One ml of hexane was added followed by 2 ml of distilled water. The tube was then shaken vigorously for 1 min and centrifuged (MSE Harrier 15/80, MSE Ltd., United Kingdom) for 3 min at 2500 rpm.

Samples were then analysed using a GC-FID (GC 14-B, Shimadzu, Japan). Separation was performed with an FFAP-polar capillary column (30 m × 0.32 mm internal diameter, 0.25 μm film thickness). Hydrogen was used as a carrier gas. After injection at 60°C, the oven temperature was raised to 150°C at a rate 40°C min^−1^, then to 230°C at 3°C min^−1^, and finally held constant for 30 min. The flame ionization was held at 240°C. Peaks were identified by comparing their retention times with those of authentic standards (Supelco Inc., Sigma-Aldrich, USA). Fatty acids were designated as an n:pωx, where n is the number of carbon atoms in the aliphatic chain, p is the number of double bonds and x is the position of the first double bond from the terminal methyl group. The analytical precision for samples was generally <5% for each sample replication.

### Data analyses

Fatty acid concentrations (mg g^−1^ dry weight) were calculated by comparing the peak area of fatty acid in the sample with the peak area of internal standard. The percentage for each fatty acid was converted from the area of chromatogram peaks. The composition is expressed as percentage of total fatty acids (Table 2).

Differences between data were analysed using the Mann–Whitney *U*-test. Differences among data were also examined using the Kruskal-Wallis test while using the Mann–Whitney *U*-test for post hoc two-group comparisons. The significance of the correlation coefficient and the regression slope were determined using a *t*-test [41].
